# Reconsidering the Use of Minocycline in the Preliminary Treatment Regime of Rheumatoid Arthritis

**DOI:** 10.7759/cureus.5351

**Published:** 2019-08-08

**Authors:** Milad Heydari-Kamjani, Michelle Demory Beckler, Marc M Kesselman

**Affiliations:** 1 Osteopathic Medicine, Nova Southeastern University Dr. Kiran C. Patel College of Osteopathic Medicine, Fort Lauderdale, USA; 2 Immunology, Nova Southeastern University Dr. Kiran C. Patel College of Osteopathic Medicine, Fort Lauderdale, USA; 3 Rheumatology, Nova Southeastern University Dr. Kiran C. Patel College of Osteopathic Medicine, Fort Lauderdale, USA

**Keywords:** microbiome dysbiosis, rheumatoid arthritis, antibiotics, minocycline

## Abstract

Strong epidemiologic, clinical, and basic science studies have identified a number of factors that may lead to rheumatoid arthritis (RA) onset and progression, particularly involving the complex interplay between genomics, environmental risk factors, the breakdown of immune self-tolerance, and microbiome dysbiosis. A chronic state of inflammation established by infectious agents has long been suspected to set the stage for the development of RA. The purpose of this article is to review the contribution of the gut, lung, and oral microbiomes to the pathogenesis of RA and consider the importance of supplementing the preliminary treatment regime of RA patients with antibiotics, in particular, minocycline. Minocycline has been used in the treatment of RA due to its bacteriostatic, as well as immunomodulatory and anti-inflammatory properties. Ultimately, a short course of antibiotic treatment with minocycline may eliminate pathogenic organisms contributing to the development and progression of RA.

## Introduction and background

Rheumatoid arthritis (RA) is a common autoimmune condition that is present in approximately 1% of the world's population [1]. The prevalence of RA in the United States is estimated to affect approximately 1.28 - 1.36 million adults [2]. Patients with RA typically present with symmetric polyarthritis affecting smalls joints of the hands and feet [1], but patients can also develop extra-articular manifestations, including diabetes, osteoporosis, vasculitis, interstitial lung disease, and cardiovascular disease [3]. The pathogenesis of RA remains unclear despite inroads into understanding the underlying mechanisms of the disease activity. Strong epidemiologic, clinical, and basic science studies have identified a number of factors that may lead to disease onset and progression, particularly involving the complex interplay between genomics, environmental risk factors, breakdown of immune self-tolerance, and microbiome dysbiosis [4].

A chronic state of inflammation established by infectious agents has long been suspected to set the stage for the development of RA. Recent studies have shown an association between the gut, lung, and oral microbiomes and their contribution to the pathogenesis of RA [5-13]. Under homeostatic conditions, the resident microorganisms at these mucosal sites establish a mutualistic relationship amongst themselves and with their host. When there is a disruption in the diversity of these organisms, a prolonged inflammatory response can occur [5]. Specifically, particular bacterial products, such as pathogen-associated molecular patterns (PAMPS), can bind to toll-like-receptors (TLR) on the surface of epithelial cells and stimulate a signal transduction cascade that results in upregulation of proinflammatory cytokines, including interleukin-1, interleukin-6, and tumor necrosis factor-alpha [14]. In addition, dendritic cells acting as antigen-presenting cells (APC) can display bacterial-induced citrullinated peptides on their major histocompatibility (MHC) complex class II molecules [15]. Protein citrullination is a post-translational modification process that occurs widely in cell differentiation, inflammation, and apoptosis [16]. This physiological process requires the presence of peptidyl-arginine deiminase (PAD) that converts C-terminal arginine residue (positive charge) to citrulline molecule (neutral charge), which creates a new epitope that is immunogenic in RA patients due to loss of self-tolerance [16]. Currently, the presence of anti-citrullinated protein antibodies (ACPAs) remains the most specific serological marker used in the diagnosis of RA [17]. Given that a subset of RA patients are genetic carriers of human leukocyte antigen (HLA)-DR alleles which encodes the MHC complex, it is plausible to hypothesize that a mutation in the MHC class II cells, together with microbiome dysbiosis, may set the stage for the development of RA [1]. Consequently, the gut, lung, and oral microbiomes likely play an important role in determining the overall tone of the immune system at these mucosal sites and are currently the leading targets for future interventions in RA [18]. The purpose of this article is to discuss the potential contributions of the gut, lung, and oral microbiomes to the pathogenesis of RA and to consider the addition of minocycline to the preliminary treatment regime of RA in efforts to eliminate pathogenic bacteria that may contribute to the development and progression of RA. 

## Review

The role of gut microbiota in RA

The potential role of the gut microbiota in the development of RA is supported by studies in animal models and analyses of the intestinal microbiome. Specifically, murine arthritis models have shown that injection of *Streptococcus pyogenes *and *Lactobacillus casei *cell wall components can induce erosive polyarthritis [19-20]. Similarly, gnotobiotic mice (germ-free mice) inoculated with segmental filamentous bacteria (SFB) results in the production of autoantibodies and development of erosive arthritis [21]. Furthermore, colonic tissue analysis of RA patients has identified 21 citrullinated peptides [8]. In particular, three of these citrullinated proteins (vimentin, fibrinogen-alpha, and actin) are known targets for ACPA, supporting the hypothesis that a breakdown in self-tolerance at the colonic mucosal site could initiate ACPA production in RA patients [8]. Lastly, fecal analysis of RA patients reveals a difference in the composition of the gut microbiome when compared to healthy individuals. For example, early RA patients appear to have a higher abundance of *Prevotella copri (P. copri​​​​​) *[9, 22]. *P. copri *has been shown to induce CD4+ T-cells to differentiate into T-helper 17 (Th17) cells resulting in the production of proinflammatory cytokines and activation of B cells with consequent antibody production [9, 22]. Collectively, these findings strengthen the hypothesis that the gut microbiome may play a role in the development and progression of RA.

The role of lung microbiota in RA

The lung microbiome is another suspected mucosal site to play a role in the pathogenesis of RA. The evidence behind this idea comes from a proteomic study where two citrullinated vimentin peptides have been identified in the bronchial and synovial tissues of patients with RA [6]. In addition, before the onset of arthritis, ACPAs are present in the sputum of ACPA-positive RA patients; however, these antibodies are not present in healthy controls [5]. Similarly, high-resolution computed tomography has shown lung parenchymal abnormality prior to the onset of the disease in ACPA-positive patients. Although the precise mechanism is unclear, smoking, a well-known risk factor for RA, has been shown to increase the citrullination of proteins by upregulating PAD [23]. As a result, citrullinated proteins can become auto-antigens in a genetically susceptible patient and may predispose an individual to develop RA.

The role of oral microbiota in RA

The oral microbiome has received a tremendous amount of attention due to the association identified between periodontitis (PD) and RA [10-13]. PD is an infectious disease caused by various oral anaerobic bacteria resulting in the destruction of the tooth-supporting structures. Specifically, two periodontal pathogens, *Porphyromonas gingivalis *(Pg) and *Aggregatibacter actinomycetemcomitans* (Aa), have been shown to produce citrullinated peptides and have been suggested to contribute to the pathogenesis of RA. Pg is gram-negative obligate anaerobic bacteria that express bacterial PAD, which is known to directly citrullinate human protein peptides at the arginine residue [24]. Aa is gram-negative facultative anaerobic bacteria that express leukotoxin A (LtxA), which destabilizes cell membrane integrity by forming pores, ultimately leading to unregulated calcium influx [25]. In neutrophils, an influx of calcium ions results in the activation of endogenous PAD, leading to indirect hypercitrullination of proteins. Combined, these examples highlight the similarities in the pathogenesis of PD and RA and provide a link between the human microbiome and the development of RA.

The role of tetracyclines in RA

The vast amount of research analyzing the gut, lung, and oral microbiomes have helped portray a convincing picture that microbiome dysbiosis at these mucosal sites can contribute to a chronic inflammatory state, the underlying mechanism in RA, and its two well-known comorbidities, coronary artery disease (CAD) [26], and cancer [27]. Given that treatment goals in RA are aimed to slow disease progression and reduce its associated comorbidities, we believe it is time to reevaluate and consider the importance of supplementing the preliminary treatment regime of RA patients with antibiotics, in particular, minocycline. Ultimately, a short course of antibiotic treatment with minocycline may eliminate pathogenic organisms contributing to the development and progression of RA. 

Tetracycline and its derivatives (minocycline and doxycycline) are a class of broad-spectrum antibiotic compounds that have in common a four-fused hydrocarbon ring. The distinguishing factors of these subclasses from one another are distinctive functional groups attached to different sites on the hydrocarbon backbone [28]. These functional groups affect the pharmacokinetic properties of these antibiotics without altering their function [28-29]. Tetracyclines function as a bacteriostatic antibiotic by specifically binding to bacterial 30S ribosomal subunits, thus preventing bacterial protein synthesis [28]. In addition to their bacteriostatic property, tetracyclines also have several inherent immunomodulatory properties [30-32]. Specifically, these drugs can inhibit phospholipase A-2, resulting in decreased production of arachidonic acid metabolites that are the key mediators of inflammation [30]. They have antioxidant properties because they can act as scavengers of hypochlorous acid [32]. Lastly, they have been shown to inhibit metalloproteinases which play a role in cartilage degradation [31]. Given these properties and their potential in modulating RA-induced inflammation, joint destruction, and arthritis, a number of clinical trials assessing tetracycline use in the treatment of RA have been performed [33-43].

Clinical trials evaluating the therapeutic effects of tetracyclines

The tetracycline class of antibiotics has been widely studied in the treatment of RA, with varied outcomes and its use remaining controversial in clinical practice. Specifically, four double-blind randomized clinical trials have been published highlighting the significance of minocycline in the treatment of RA patients. The first study involved 80 long-term RA patients who did not benefit from more than one disease-modifying antirheumatic drug (DMARD) [33]. Patients were treated with 200 mg of oral minocycline or a placebo in conjugation to their standard therapy for six months [33]. Compared to the control group, the treatment group showed a statistical difference in laboratory parameters, such as erythrocyte sedimentation rate (ESR), C-reactive protein (CRP), and IgM rheumatoid factor (RF) levels [33]. In a second study, 219 adults with active RA who had limited benefits using DMARDS were examined [34]. Those patients were treated with 200 mg of oral minocycline or placebo for 48 weeks. The minocycline treatment group showed statistically significant improvement in joint swelling and joint tenderness, ESR, and RF levels. The last two studies were conducted by the Rheumatoid Arthritis Investigational Network (RAIN) Group [35-36]. Their first study involved 46 naive RA patients that were diagnosed for less than one year [35]. Patients received 100 mg of oral minocycline twice daily for six months and were compared to the placebo group. The treatment group showed a statistically significant improvement in the morning stiffness, patient global status, and physician global status. In the second study by the RAIN Group, 60 RA patients with a duration of less than one year were randomized to receive 100 mg of oral minocycline twice daily or a placebo [36]. All patients were on low-dose prednisone and were never treated with DMARDS. The patients treated with minocycline were more likely to achieve greater than 50% improvement in the American College of Rheumatology (ACR) criteria and did so while receiving less prednisone. In addition to the four double-blind studies, two open-label trials showed similar positive results when evaluating the efficacy of minocycline in the treatment of RA [37-38]. The first study included 18 RA patients who received 200 mg of oral minocycline daily for 48 weeks [37]. The second study included 10 RA patients who received 400 mg of oral minocycline for 16 weeks [38]. Both studies showed statistically significant improvement in the Disease Activity Score-28 (DAS-28). While minocycline showed some benefit, the combination of tetracycline [39] and doxycycline [40-42] showed no benefit with studies demonstrating no statistically significant difference in reducing DAS-28 when comparing RA patients to those who received a placebo. These clinical trial studies were further analyzed by a meta-analysis highlighting that minocycline can significantly reduce the DAS-28 score in RA patients with no significant effect on radiographic progressions [43]. Combined, these clinical trials suggest that tetracycline (in particular, minocycline) appears to be beneficial in reducing RA symptoms.

Previous clinical trials have analyzed the overall therapeutic effects of tetracycline in RA patients, but they did not differentiate whether it is through its bacteriostatic or immune-modulatory properties [33-43]. However, a recent case presentation demonstrated that a virulent strain of Aa initially led to subacute endocarditis, which later resulted in the development of early seropositive RA in a genetically susceptible individual [44]. Perhaps more importantly, switching treatment options from immunosuppressive agents (methotrexate, prednisone, etanercept, leflunomide) to an antibacterial agent (intravenous ceftriaxone) for six months resolved the RA symptoms characterized by normal levels of CRP and ACPA [44]. Although tetracyclines were not used in this particular case, they have shown in vitro to be effective against Aa JP2, the most virulent type of this bacteria [45]. Given this, it may be plausible that using tetracyclines could have shown similar results. This case presentation further adds to the theory that microbiome dysbiosis in a genetically susceptible individual can result in the production of immunologic response to naïve peptides and set the stage for the development of RA. Therefore, we believe it is imperative for clinicians to assess for infection etiology as a source of chronic inflammation when a patient presents with symptoms of RA. Since the treatment goal is to slow disease progression and reduce its associated comorbidities, it is time to reevaluate the efficacy of antibiotics and consider their addition to the preliminary treatment regime of patients with RA. Ultimately, a short course of antibiotic treatment with minocycline may eliminate pathogenic organisms contributing to the development and progress of RA. 

Side effects of tetracyclines

Minocycline is typically a well-tolerated antibiotic; however, common side effects can be seen in up to 18% of patients [46]. These include photosensitivity, hyperpigmentation, dizziness, nausea, headache, tinnitus, diarrhea, and abdominal pain [46]. There are several severe side effects of minocycline that are worth addressing. First, it has been reported that individuals with abnormal liver function may develop drug-induced lupus [47]. However, upon discontinuation of the medication, the symptoms will spontaneously resolve with a few weeks to months. Second, there appears to be a lower risk of *Clostridium difficile *infection compared to clindamycin that has a well-known increased risk of infection [48]. Third, the use of chronic tetracyclines may be associated with an increased risk of developing breast cancer in women or prostate cancer in men [49-50].

## Conclusions

Rheumatoid arthritis is an autoimmune condition that involves a complex interplay of multiple factors, including microbiome dysbiosis. The gut, lung, and oral microbiome dysbiosis may play a role in the pathogenesis of RA by contributing to a chronic inflammatory state. Given our current understanding of the microbiome, the addition of tetracyclines antibiotics, in particular, minocycline, may pose benefits through its bacteriostatic and immunomodulatory properties. Ultimately, a short course of antibiotic treatment with minocycline may eliminate pathogenic organisms contributing to the development and progression of RA. 
